# Autobiographical Case Report: A Rubber Band, a Glass of Orange Juice

**DOI:** 10.7759/cureus.18939

**Published:** 2021-10-21

**Authors:** Leonard H Sigal

**Affiliations:** 1 Rheumatology, Rutgers Robert Wood Johnson Medical School, New Brunswick, USA

**Keywords:** myocardial infarction, shock gall bladder, survival, ards, band hemorrhoidectomy, septic shock

## Abstract

Seemingly simple procedures can go desperately wrong. Physicians are used to "knowing" and "being in charge". When a physician is suddenly the profoundly ill patient, the inversion of roles can be frustrating, frightening, and disorienting.

## Introduction

Experience with personal or family illness can enhance insights and empathy in healthcare professionals. Not that I am suggesting young physicians go looking for potentially deadly disorders to acquire, but knowing what it is like to be on the other end of the stethoscope can be enlightening.

## Case presentation

I had hemorrhoids, itching, and bleeding, an annoyance, an embarrassment. The colorectal surgeon suggested banding: a rubber band around the hemorrhoid’s base causes the 'rhoid to necrose and fall off, along with the rubber band. Simple.

I had the procedure on Wednesday. Two days later, a lovely Friday in late April, I delivered Rheumatology Grand Rounds at a nearby medical school. It was an opportunity to meet members of the faculty whom I had known only as clinical and laboratory giants. As I drove home, I began experiencing dizziness and headache, like a viral syndrome. There were moments where I felt lightheaded and my vision spun, my head and neck not following the same script. I got home and had more of these episodes, each lasting less than a second.

There was a temperature of 103 the following morning, although nothing GI or respiratory, no myalgias or headache. I stayed in bed except to prepare a grilled cheese lunch for my daughter and her playdate friend; intermittently feeling faint. I skipped lunch and dinner - simply no appetite - but felt I needed something in my stomach so I went downstairs to get a glass of orange juice and then to sleep.

At about 2 am, my wife heard a thud in our bedroom, between the bed and the bathroom. That thud was me; I had been awakened by indigestion and got up to get a glass of water. About two-thirds of the way to the bathroom I collapsed (might have been twice) and awoke on the floor, shivering in the cool room, with my wife leaning over me. I did not know what had happened; I could move all four limbs but had a tingling sensation in my upper lip. Against all odds, I thought I had had a stroke, took an aspirin, and went to bed. I called a neurology colleague from the Medical School. He suggested going to the nearest ER.

No “911” call, we drove the one mile to our local hospital and walked to what we thought was the entrance to the ER only to find the door locked. So, we walked around the building to enter the ER. The ER staff determined my BP was 70/50. Then things started happening fast, as my BP dropped to 50/30. Rapidly into Trendelenburg (as they discuss this I quip, “Yup, I think Trendelenburg would be a really good idea”), two femoral lines, a Swan-Ganz, a urinary catheter, EKG leads. I was wisecracking the entire time to compensate for my fear. One of the ER docs said, “You know we don’t usually have conversations with people with your BP!”

I was moved upstairs to the ICU. BP stabilized on pressors, fluids, and broad-spectrum antibiotics. With so much monitoring noise and lights all night, there was really no way to get any sleep. Muhlenberg residents trickle in; my students the past year on the rheumatology elective, having seen my name on the board, drop in. My primary care physician (PCP) just happens to be in the hospital that morning and also sees my name on the board, so he takes charge. But there was no explanation for what happened; most of that morning is a blur of activities, monitoring, confusion, and fear.

I am transferred to my own academic medical center (lights, no siren) and enter my second ICU. There was more evaluation by residents who had trained with me, and by medical and surgical colleagues. I now have a pleural effusion and acute respiratory distress syndrome (ARDS), ileus, high fever, an elevated creatine kinase-MB (CPK-MB), and “shock gallbladder”; poor design giving the gallbladder only one artery! Still no explanation!

My daughters and wife come to visit me that afternoon. My younger daughter, aged six years, will NOT cross the threshold of my room; she is gripping my wife’s leg in terror and will not move. Meanwhile, I am crushing the hand of the poor nurse standing by me as I try desperately not to cry. I must look a fright, with a tube in my neck and in my groins, pale, weak, frightened, but I feel I must be strong, I must be DADDY.

Inside I am scared to death (nearly), the terror of the unknown enveloping me. Of looking weak to my family. Of having bits of me die. Of being crippled. Of dying. Of leaving my family alone. Of not seeing my girls grow up. Of never being “me” again. My father was an old, weak man; am I to become frail and fragile...and pathetic?

I have polymicrobial Gram-negative sepsis, but why? There is talk of removing my gallbladder and my rectosigmoid, now riddled with infection, meaning a bag for the rest of my life? Nope, no way, not gonna happen; I cannot accept such an alteration of my body. I came here with these organs and they will be in situ when I leave! I am short of breath, ARDS not heart failure. The only good news is that I have survived to this point. No one can explain this sudden devastating illness. Am I dying? No one really knows. “The next few hours…”.

I am stabilized and some of the lines are removed (by this time my 10-year-old daughter knows to call the thing in my neck a Swan-Ganz). I am transferred to a room on the medical ward. My wife comes to visit and we chat. Sedation with morphine takes me on a vacation I had always meant to take, a canal barge in the south of France. Trees overhanging the canal, we putt along and then stop, tie off, and I ride the blue bicycle sitting just aft of the cabin into the next village for a baguette. That journey is interrupted by returns to our chat, but each time I nod off I return to that bicycle and barge…and baguette. The vacation was nice, but I find the inability to concentrate annoying so we downshift to a lesser drug.

Later that day, my colorectal surgeon finally offers an explanation: the rubber band snapped off prematurely before the hemorrhoid had died and sealed off; luminal contents poured into the wound and so I have transmural colitis and sepsis. Makes sense. I am told that the odds are 1 in 10,000…it figures! [1]

Later it occurs to me that the orange juice saved my life. With no reflux, no awakening, no collapse, and no ER, perhaps my wife would have awakened the following morning next to a corpse!

My pleural effusion must be evaluated. A resident who had just finished our elective, with a rather tepid evaluation from me comes in. I looked at her, then the syringe, then her again. “Were you satisfied with your evaluation?”, I ask. She gives me a sheepish smile and says, “Yes, Dr. Sigal”. OK, let’s do this, said I. It was an uneventful procedure and fluid. A few days later, I notice a pain in my back; the same place as the needle. OK, I finally realize the connection.

Antibiotics, decreased IV fluid, some oral intake, walking around the ward a few times, stabilizing myself on the wall and a nurse’s arm, then only on the wall, then slowly by myself. Each step was a victory, each lap a triumph. I was discharged with a catheter in my non-functioning, but not dead, gall bladder. I drain bile from the attached bag a few times a day, a very strange experience.

The outpatient stress test is normal and EKG reveals no changes. It was one more step to being healthy again. Small but significant progress day by day. I realize that I am feeling woozy most of the time and very postural. My pulse is 35, so the post-MI beta-blocker is discontinued.

Getting out of bed weeks later, a kink in the catheter draining my still nonfunctioning gallbladder causes me instantaneous 12 out of 10 pain. I cannot breathe, I cannot move. I whisper to my wife who was in the bathroom who finally hears me. Off to the ER; up to the OR, where the radiologist replaces the catheter. During the procedure, my pulse dips to 30. He tells me NEVER to come back again!

Two months later, the catheter is removed, the small wound heals and I begin to feel more normal. Strength and activities increase. It is only in September, four months after my illness, while hiking at Rocky Mountain National Park at more than 8,000 above sea level that I finally have proof that I am “back”. But it takes me well over a year before I can drink orange juice again.

I was on the other end of the stethoscope 21 years ago. I felt fear and hopelessness and doubt and helplessness and confusion and anger and oh so vulnerable. I simply could not appear weak and impotent to my daughters. I froze at even thinking of my own death, leaving a widow and two young girls who would remember me as only a distant and fading presence; I could not cope with thoughts of being vulnerable and weak and impotent for the rest of a diminished life.

My thanks to the house staff and faculty at Muhlenberg Hospital and R W Johnson Medical Center for saving my life and to my wife, Barbara, for being there with me at all times, and to my daughters for finally accepting that their Daddy was really back.

## Discussion

Early on with no answers, there could be no reassurance, no specific and targeted strategy, no promise of a cure. As a logical thinker and teacher, an attending physician, I wanted facts and explanations and plans and I wanted them now. There was talk of major surgery that was beyond disheartening, threatening my very core. I was impatient. I had an MD after my name and could think (occasionally) clearly, but could not grasp what was going on or be reassured until there was a concrete answer and the possibility of a return to normal function. I experienced a “surgical failure,” near death, cataclysmic consequences, complications, and a slow recovery. I was entirely “out of control”, anathema to any physician, and I was totally dependent upon others to take care of, support, and inform me. Upon discharge, every day was filled with doubt about my future. Even now I wonder about the status of the cells in my now regenerated rectosigmoid.

I was given the best care possible. Answers were in a language I could understand. Friends and colleagues showed their concern. I was surrounded by care and empathy. We can and must try to understand how patients feel, during the hospital stay when they experience pain and confusion, and uncertainty. Communication in a language they understand, using words they understand, and being sensitive to their needs are of utmost importance for a treating doctor.

## Conclusions

I cannot claim to have been transformed by this near-death experience, but I do think I became more aware and sensitive to the needs of my patients and their families, a better listener, a better communicator. I think I became more empathic. I think I became a better doctor. At least that is what it feels like as I now hang up my stethoscope for the last time.
